# The effect of urban safe community implementation on the reduction of traffic accidents from 2004 to 2017 in the Municipality of Zarand 

**Published:** 2019-08

**Authors:** Mashaallah Arabpour, Hadi Zarei, Vahidreza Borhaninejad, Zahra Nekoei

**Affiliations:** ^*a*^Department of Environmental Management, Islamic Azad University, Meybod, Iran.; ^*b*^Department of Health Services, Kerman University of Medical Sciences, Kerman, Iran.; ^*c*^Department of Environmental Health, Zahedan University of Medical Sciences, Zahedan, Iran.

## Abstract

**Background:**

This research aims at exploring and recognizing the effectiveness of implementing a community-based safety plan on reducing the occurrence of road accidents in the city of Zarand.

**Methods:**

The research method is descriptive method with analytical approach. To analyze the obtained statistical data, descriptive and inferential statistics were used. For field surveys, a questionnaire was used and for analyzing the data obtained from the questionnaire, the inferential statistics such as Friedman and Measurement tests were used. A paired T-test was used to test the significance of the hypotheses.

**Results:**

The research showed the positive effect of the implementation of safe community plan on the reduction of accidents. The main cause of the accidents was human factors, including unauthorized speed and overtaking, left-leaning and rushing. After that, the quality and technical issues of the road have been identified, and according to the interviewees, the main cause of accidents is identified as hazardous route bends, especially in rural areas, poor quality paths and inappropriate signs. The technical and qualitative factors of the car are in the third place and, finally, the environmental and natural factors are the fourth. Also, hypotheses related to the role of the implementation of the safe community plan in reducing mortality, car accidents and pedestrian accidents were confirmed. It was also found that the role of human factors was reduced by implementing strategies such as training and changing the behavior of people, the quality of roads and technical issues.

**Keywords:**

Safe society, Accidents, Human factors, Environmental factors, Road factors, Zarand City

